# Eosinophilia and parasitic infestations in patients with chronic obstructive pulmonary disease

**DOI:** 10.1038/s41598-020-69541-7

**Published:** 2020-07-27

**Authors:** Narongkorn Saiphoklang, Chanya Chomchoey

**Affiliations:** Division of Pulmonary and Critical Care Medicine, Department of Internal Medicine, Faculty of Medicine, Thammasat University, 99/209 Paholyotin Road, Klong Luang, Pathum Thani 12120 Thailand

**Keywords:** Diseases, Health care, Medical research

## Abstract

Eosinophilia may guide response to inhaled corticosteroid treatment in patients with chronic obstructive pulmonary disease (COPD). This study aimed to determine prevalence of eosinophilia and parasitic infestations in these patients. We conducted a prospective cohort study between February 2019 and January 2020 and screened 107 stable COPD patients. A total of 77 subjects (84.4% men) were included. Age was 73.8 ± 8.9 years. Forced expiratory volume in 1 s was 66.5 ± 25.5%. Smoking history was 25.9 ± 18 pack-years. Comorbidities included cardiovascular disease (57.1%). Respiratory symptoms were assessed by modified Medical Research Council dyspnea score (1.6 ± 0.8), chronic obstructive pulmonary disease Assessment Test score (9.3 ± 4.9), and 6-min walking distance (317.2 ± 135.2 m). Patients with blood eosinophil count at least 100 cells/μL were 79.2% and at least 300 cells/μL were 33.8%. Intestinal parasites were not found. Significant positive correlations were found between high blood eosinophilia and some post-bronchodilator lung function parameters. In conclusion, eosinophilic COPD was not uncommon. No intestinal parasite was found in this population. This study suggests that stool parasite exam might be omitted for routine practice.

**Clinicaltrials.in.th Number:** TCTR20191129002.

## Introduction

Chronic obstructive pulmonary disease (COPD) is characterized by persistent airflow limitation. It is commonly diagnosed in patient aged more than 40 years with chronic cough, dyspnea and/or history of exposure to risk factors for the disease. A major significant risk factor of COPD is cigarette smoking. Moreover, biomass fuel and dust particles are well known as risk factors besides smoking. According to Global Initiative for Chronic Obstructive Lung Disease (GOLD) guideline^1^, diagnosing COPD is supported by spirometry using post-bronchodilator FEV_1_/FVC ratio less than 0.7.

Assessment of blood eosinophil levels in COPD patients has an important role in COPD management. Previous studies showed the correlation between blood eosinophil level and incidence of exacerbation of the disease^2,3^. Furthermore, high blood eosinophil count (BEC) is correlated with response to inhaled corticosteroid (ICS)^4^.

There is a change of the current GOLD guideline^1^ for COPD management compared with the previous guideline. Clinical deterioration is assessed by modified Medical Research Council (mMRC) dyspnea scale ≥ 2 or COPD Assessment Test (CAT) score ≥ 10. Exacerbation history is considered as predictor of higher risk of COPD exacerbation. An exacerbation history with hospitalization in the last year or ≥ 2 moderate exacerbations per year with BEC ≥ 100 cell/µL should be prescribed inhaled corticosteroid plus long-acting beta-agonist (ICS/LABA)^5^. Moreover, COPD patients with an exacerbation and BEC ≥ 300 cell/µL are a significant prognosticator of better response to ICS^6^. However, eosinophilia results from several etiologies such as parasitic infections, allergy, hematologic malignancies, and autoimmune diseases. The most common cause of high BEC in developing countries is parasitic infestation^7^. In this study, we aimed to determine the prevalence of intestinal parasitic infestation in COPD patients with blood eosinophilia.

## Methods

### Study design

A prospective cohort study was conducted at Thammasat University Hospital in Thailand from February 2019 through January 2020. Stable COPD patients aged 40 years or older with smoking history more than 10 pack-years were recruited from COPD clinic. Exclusion criteria were history of COPD exacerbation in the past 3 months, other chronic respiratory diseases such as interstitial pulmonary fibrosis or bronchiectasis, inability to perform spirometry, inability to perform stool examination and complete blood count (CBC), inability to walk, diagnosis of asthma-COPD overlap (ACO), undergoing tracheostomy tube, use of mechanical ventilation, and treatment with systemic corticosteroid in the past 6 weeks. Patients’ prescriptions were checked before every CBC measurement to ensure that all eosinophil counts were not affected by systemic corticosteroids.

Ethic approval was obtained from the Ethics Committee of Faculty of Medicine, Thammasat University (IRB No. MTU-EC-IM-1-265/61), in compliance with Declaration of Helsinki, The Belmont Report, CIOMS Guidelines and The International Practice (ICH-GCP). All methods were performed in accordance with these guidelines and regulations. All participants provided written informed consent.

### Procedures

Patients demographic data, clinical characteristics, comorbidities, mMRC, CAT scores, vital signs, current medications, exacerbation history and vaccinations were recorded. CBC and stool samples were collected at the first date of the study and one week later. The stool test was done by simple smear method with direct saline stool microscopy for parasites. Stools were examined to detect common intestinal parasite species in each of two groups: helminths (echinostome, *Gnathostoma spinigerum,* hookworms*, Opisthorchis viverrini*, *Strongyloides stercoralis, Taenia* spp., *Trichuris trichiura*) and protozoa (*Balantidium coli*,* Blastocystis hominis*,* Endolimax nana*,* Entamoeba coli*,* Entamoeba dispar*,* Entamoeba histolytica*,* Giardia intestinalis, Iodamoeba bütschlii*, *Sarcocystis hominis*)^8–11^. Six-minute walk test (6MWT) and standard pre- and post-bronchodilator spirometry according to ATS/ERS guideline^12^ were performed in the first visit.

The patients were followed up 3 months later to evaluate respiratory symptoms and signs, perform spirometry and 6MWT for 6-min walking distance (6MWD) for assessment as well as CBC.

### Outcomes

The primary outcome was the prevalence of parasitic infestation in COPD patients. Definition of parasitic infestation was presence of adult parasitic forms, larvae or ova in stool. The secondary outcome was the high BEC prevalence in COPD patients. We defined high blood eosinophils as 100 cells/µL or more.

### Statistical analysis

Based on a previous study^13^, prevalence of high BEC (> 150 cells/µL) in COPD was 20%. The sample size was calculated using 80% power, 5% type I error, and 10% precision margin. Thus, the sample size would be 62.

Statistical analyses were performed using SPSS version 20.0 software (IBM Corp., Armonk, NY, USA). Chi-squared test was used to compare categorical variables between two groups. ANOVA was used for comparison of 3 visits. Pearson correlation were used for correlation analysis between groups. A two-sided p-value < 0.05 was considered statistically significant.

## Results

**Figure 1:**
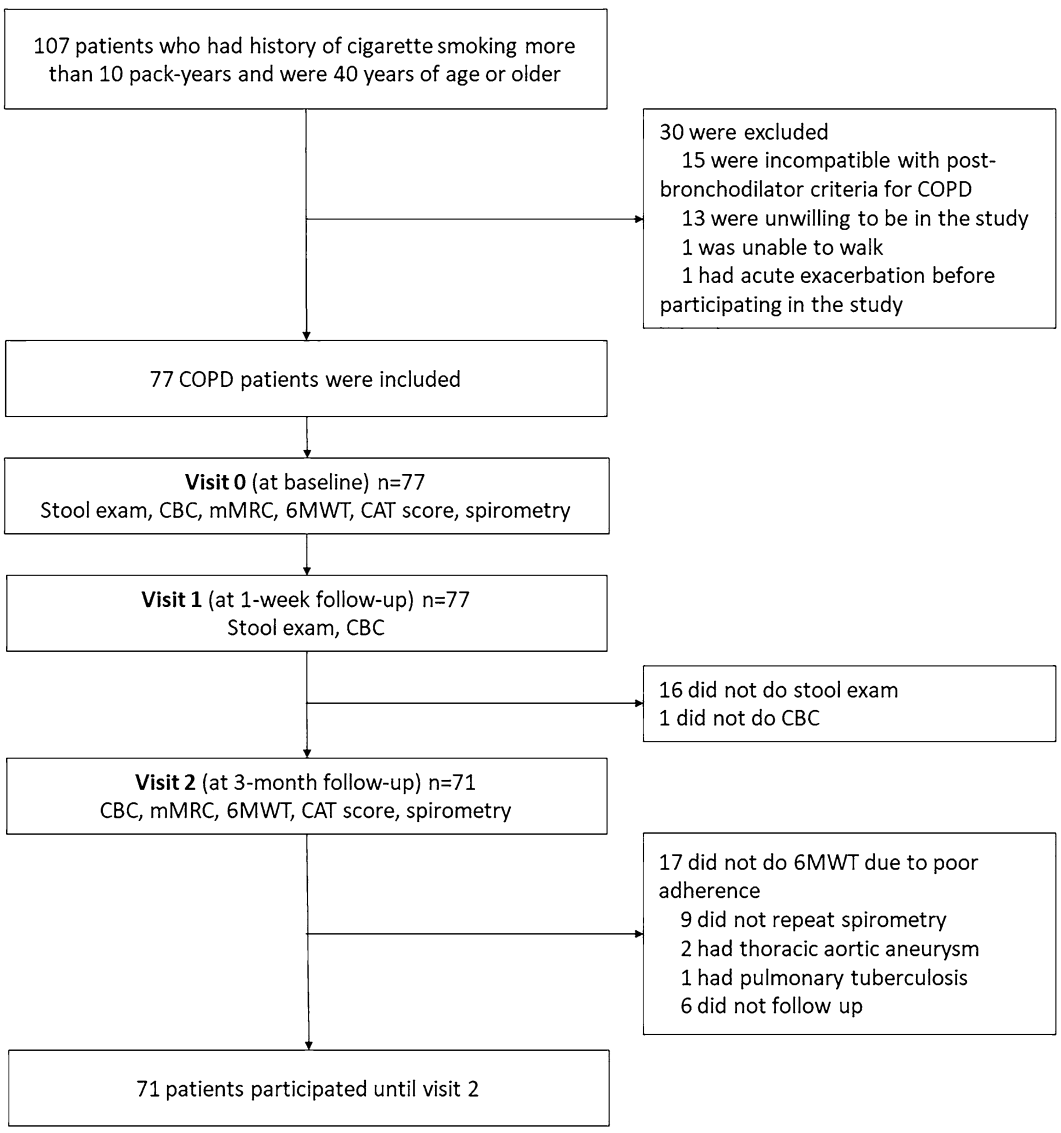
Flow chart of patient recruitment to the study. *COPD* Chronic obstructive pulmonary disease, *mMRC* modified Medical Research Council, *CAT* COPD Assessment Test, *CBC* complete blood count, *6MWT* Six-minute walk test.

A total of 107 COPD patients were recruited. Of these, 77 patients were eligible for inclusion, however 30 patients were excluded (Fig. 1). At the end of the study, there were 71 patients who still participated. Mean age of the participants was 73.78 ± 8.91 years. Mostly, they were male. Mostly the participants were classified as COPD group B and were in grade 2 by spirometry classification. Cardiovascular disease was the most common of comorbidity. Moreover, common medications included long-acting muscarinic antagonists and LABA/ICS. Postbronchodilator FEV_1_/FVC ratio was 58.64 ± 11.22. Generally, they did not have significant bronchodilator response by the evidence of FEV_1_ and FVC change (Table 1).

**Table 1:** Demographic and baseline characteristics of COPD subjects.

Characteristics	N = 77
Age, years	73.78 ± 8.91
Male	65 (84.4)
BMI, kg/m^2^	23.40 ± 4.40
Smoking, pack-years	25.9 ± 17.97
Active smoking	8 (10.4)
**GOLD classification**	
A	28 (36.4)
B	31 (40.3)
C	5 (6.5)
D	13 (16.9)
**Spirometry grading**	
1	25 (32.5)
2	31 (40.3)
3	16 (20.8)
4	5 (6.5)
**Clinical history**	
AECOPD in last 1 year	22 (28.6)
AECOPD with hospitalization in last 1 year	18 (23.4)
Home oxygen supplementation	1 (1.3)
Oxygen saturation, %	96.82 ± 1.52
**Medication**	
Inhaled short-acting bronchodilator	55 (71.4)
Inhaled long-acting muscarinic receptor antagonist	39 (50.6)
Inhaled long-acting beta2 agonist	4 (5.2)
Inhaled long-acting muscarinic receptor antagonist plus long-acting beta2 agonist	11 (14.3)
Inhaled corticosteroid plus long-acting beta2 agonist	39 (50.6)
Oral bronchodilator	5 (6.5)
Phosphodiesterase-4 inhibitor	3 (3.9)
Leukotriene receptor antagonist	7 (9.1)
Xanthine derivative	18 (23.4)
Azithromycin	4 (5.2)
**Vaccination**	
Influenza vaccine within 1 year	55 (71.4)
Pneumococcal vaccine	39 (50.6)
**Comorbidity**	
Hypertension	44 (57.1)
Benign prostatic hyperplasia	16 (20.8)
Allergic rhinitis	14 (18.2)
Stroke	12 (15.6)
Coronary artery disease	9 (11.7)
Chronic kidney disease	7 (9.1)
Obstructive sleep apnea	4 (5.2)
Lung tumor	4 (5.2)
Congestive heart failure	2 (2.6)
**Spirometric data**	
Post-bronchodilator FVC, L	2.39 ± 0.79
Post-bronchodilator FVC, % predicted	80.68 ± 19.12
FVC change after bronchodilator, %	7.65 ± 12.82
Post-bronchodilator FEV_1,_ L	1.42 ± 0.58
Post-bronchodilator FEV_1,_ %predicted	66.49 ± 25.53
FEV_1_ change after bronchodilator, %	9.08 ± 11.71
Post-bronchodilator FEV_1_/FVC, %	58.64 ± 11.22
**Functional performance**	
mMRC	1.58 ± 0.81
CAT score	9.28 ± 4.89
6MWD	317.15 ± 135.25

Data shown as mean ± SD or n(%).

*BMI* body mass index, *kg* kilogram, *m* meter, *AECOPD* acute exacerbation of chronic obstructive pulmonary disease, *FVC* forced vital capacity, *L* liter, *FEV1* forced expiratory volume in 1 s, *mMRC* modified Medical Research Council, *CAT* COPD Assessment Test, *6MWD* 6-minute walking distance.

No parasite was found by stool exam. All COPD patients had no symptoms of intestinal parasites including abdominal pain, diarrhea, vomiting, constipation, skin rashes or eczema, anemia, or unexplained weight loss.

CBC data including BEC was not different in 3-month interval (Table 2). At baseline, BEC ≥ 100 cells/µL was found in 61 patients (79.2%), BEC ≥ 300 cells/µL was found in 26 patients (33.8%), and BEC ≥ 2% was found in 53 patients (68.8%). There was significant bronchodilator response by evidence of FEV_1_ change in the high BEC group (Table 3).

**Table 2:** Comparison in complete blood count results between baseline, 1-week and 3-month follow-up.

Variables	Baseline	1-week follow-up	3-month follow-up	P-value
Hemoglobin, g/dL	12.89 ± 1.75	12.87 ± 1.81	12.96 ± 1.48	0.293
White blood cell count, cells/µL	7,344.00 ± 2,646.27	6,818.05 ± 2,157.15	7,610.91 ± 2,821.79	0.13
Neutrophil, %	61.07 ± 11.04	57.92 ± 9.24	61.72 ± 12.93	0.27
Monocyte, %	8.97 ± 3.01	9.46 ± 4.19	8.66 ± 2.35	0.743
Lymphocyte, %	24.99 ± 9.51	27.01 ± 8.87	24.66 ± 10.42	0.555
Eosinophil, %	4.13 ± 3.99	4.92 ± 5.06	4.12 ± 4.39	0.072
Basophil, %	0.67 ± 0.38	0.69 ± 0.39	0.68 ± 0.41	0.483
Platelet, /µL	238,146.67 ± 67,108.16	240,175.68 ± 67,361.30	236,592.59 ± 83,024.10	0.816
BEC, cells/µL	290.29 ± 290.20	297.85 ± 390.71	295.31 ± 386.35	0.192

Data shown as mean ± SD, ANOVA was used for comparison of 3 visits.

*BEC* blood eosinophil count.

**Table 3:** Characteristics of COPD patients with or without blood eosinophil count ≥ 300 cells/µL.

Characteristics	BEC < 300 cells/µL (N = 49)	BEC ≥ 300 cells/µL (N = 26)	P-value
Male	41 (83.7)	22 (84.6)	0.82
Active smoking	7 (14.3)	1 (3.8)	0.33
AECOPD in last 1 year	15 (30.6)	6 (23.1)	0.63
AE hospitalization group	14 (28.6)	4 (15.4)	0.32
Home oxygen therapy	1 (2)	0 (0)	0.75
**Medication**			
Inhaled shorting-acting bronchodilator	32 (65.3)	21 (80.8)	0.25
Inhaled long-acting muscarinic receptor antagonist	27 (55.1)	11 (42.3)	0.57
Inhaled long acting beta2 agonist	2 (4.1)	2 (7.7)	0.76
Inhaled long acting muscarinic receptor antagonist plus long acting beta2 agonist	9 (18.4)	1 (3.8)	0.8
Inhaled corticosteroid plus long acting beta2 agonist	24 (49)	14 (53.8)	0.92
Oral bronchodilator	4 (8.2)	1 (3.8)	0.72
Leukotriene receptor antagonist	4 (8.2)	3 (11.5)	0.80
Xanthine derivative	13 (26.5)	5 (19.2)	0.57
Azithromycin	4 (8.2)	0 (0)	0.30
**Vaccination**			
Influenza vaccine within 1 year	36 (73.5)	18 (69.2)	0.74
Pneumococcal vaccine	25 (51)	14 (53.8)	0.34
**Comorbidity**			
Hypertension	29 (59.2)	13 (50)	0.35
Coronary artery disease	6 (12.2)	3 (11.5)	0.87
Congestive heart failure	2 (4.1)	0 (0)	0.56
Chronic kidney disease	2 (4.1)	5 (19.2)	0.09
Benign prostatic hyperplasia	12 (24.5)	4 (15.4)	0.50
Obstructive sleep apnea	3 (6.1)	1 (3.8)	0.86
Lung mass	3 (6.1)	1 (3.8)	0.86
Chronic thromboembolic pulmonary hypertension	1 (2)	0 (0)	0.75
Allergic rhinitis	7 (14.3)	7 (26.9)	0.32
Stroke	8 (16.3)	3 (11.5)	0.34
**Spirometric data**			
FVC change after bronchodilator ≥ 200 mL	12 (24.5)	11 (42.3)	0.52
FEV_1_ change after bronchodilator ≥ 200 mL	3 (6.1)	8 (30.8)	0.048
FVC change after bronchodilator ≥ 12%	10 (20.4)	8 (30.8)	0.71
FEV_1_ change after bronchodilator ≥ 12%	15 (30.6)	11 (42.3)	0.63

Data shown as n (%).

*FVC* forced vital capacity, *L* liter, *FEV*_*1*_ forced expiratory volume in 1 s, *AECOPD* acute exacerbation of chronic obstructive pulmonary disease.

Post-bronchodilator parameters were significantly higher than baseline data in 3-month interval (Table S1). In contrast, functional assessment was not different in 3-month interval (Table S2).

BEC was significantly correlated with post-bronchodilator FVC (r = 0.24, P = 0.041) and post-bronchodilator FEV_1_ (r = 0.27, P = 0.023) (Table S3) but there was no correlation between BEC and mMRC, CAT scores or 6MWD (Table S4).

## Discussion

Our study was the first prospective cohort study to determine prevalence of eosinophil and parasitic infestation. It has been shown that COPD patients in our setting were mostly in COPD grade 2 group B. ICS/LABA were used as the second most common treatment (50%).

A quarter of the subjects in our study had history of exacerbation in the previous year. Moreover, BEC at least 100 cells/µL were found commonly (79%). One-third of subjects (34%) had blood eosinophil levels of more than 300 cells/µL. From this evidence, it has been shown that high BEC was not uncommon in this Asian population. We knew that the most common cause of high blood eosinophil in Thailand was due to parasitic infestation and the most common parasite found was *Strongyloides stercoralis* in 33%^11^. Previous studies^14,15^ revealed that direct smear method for parasite exams had lower sensitivity than stool concentration techniques with formol ether or formalin-ethyl acetate concentration (12.5–61% vs 73.2–92%). However, our study was trying to improve the sensitivity by performing 2 consecutive stool exams (1-week interval). Our study found no parasites in these exams. This implied that health care and sanitation have improved in Thailand. Therefore, stool examination might be omitted in routine COPD patient care.

Previous studies of Vedel-Krogh et al.^2^ and Bafadhel et al.^3^ found that there was a relationship between high BEC and acute exacerbation of COPD (AECOPD)^2, 3^. High BEC was also related with significant ICS response as shown in the study of Brightling et al.^4^.

The study of Kolsum et al.^13^ showed positive correlation between BEC and sputum eosinophil count. Balzano et al.^16^ found that high sputum eosinophil was correlated with low percent predicted of FEV_1_ and FEV_1_/FVC ratio. However, our study did not perform sputum exam for eosinophils and did not find any correlation between BEC and AECOPD.

The study of Adir et al^17^ showed that the number of patients who had percentage of BEC ≥ 2% was 71%. In our study, the result was almost the same (69%). There were several studies of COPD patients who had high BEC and percentage of eosinophils^18–23^. Prevalence of eosinophilia (≥ 2%) in our study was almost similar to the TRISTAN study^21^. Interestingly, our finding showed more patients with BEC > 300 cells/µL (33%) than in WISDOM study (20%)^22^.

In this study, we assumed that BEC was reliable because it did not fluctuate between 3 visits. Our study also strictly excluded patients who might have taken any medication that could have affected the BEC, especially systemic corticosteroids. There was a previous study of Oshagbemi et al.^24^ which supported this data especially in high BEC group (< 340 cells/µL) who had a tendency to have more BEC stability. A study of Southworth et al.^25^ found similar results. Repeated BEC measurements at 6 months or > 2 years remained in the same range using the 150 eosinophils per μL threshold. Therefore, using the 150 eosinophils per μL threshold indicated good long-term biomarker stability.

Review of Tashkin et al.^26^ showed diverse results between lung function parameter and BEC from various prior studies. Some studies^23,27^ showed that higher BEC was related to lower pulmonary function parameters but ECLIPSE study^19^ had opposite results. In the ECLIPSE study^19^ (n = 1,483), the patients with blood eosinophil persistently ≥ 2% (n = 554) had a higher mean percent predicted of FEV_1_ than that with blood eosinophil persistently < 2% (n = 201, 51% vs 48%; P = 0.009). The outcomes corresponded to our study.

Our study showed positive correlation between BEC and post-bronchodilator FVC as well as post-bronchodilator FEV_1_. Interestingly, FEV_1_ change of 200 ml was also significant in high BEC group. This might imply that high BEC patients had a tendency toward more bronchodilator reversibility.

In addition, we found no correlation between BEC and AECOPD, or between BEC and functional performances. After 3-month follow-up, functional performances had not improved significantly as lung function had, probably because higher lung function parameters did not reach the minimal clinically important difference (MCID) FEV_1_ was changed by 80 ml in our study. As a result, the enhancing functional performances were not obviously seen.

Clinical applications are proposed from our study. Firstly, CBC might need to be checked once because our study suggested the eosinophil level was not significantly different between 1-week interval and 3-month follow-up. Secondly, lung functions, particularly in bronchodilator reversibility, should be emphasized for patients with high eosinophil levels. Lastly, stool examination might not be necessarily be routinely tested in Thailand for COPD patient care.

Our study had a few limitations. Firstly, because of the small size of population, some results might not be obviously different between groups. Because this study was held in a single research center in Thailand, the result might not be applicable to other ethnicities or countries. Secondly, data on medication adjustment was not collected throughout the 3-month interval. Therefore, we could not assume that improvement of post-bronchodilator lung functions were solely as a result of high BEC, but this might be from some medications that were added to the treatment regimen. Lastly, due to limitation of direct stool exam, and because we did not do stool concentration methods for parasites, some parasites might not have been detected.

## Conclusions

Eosinophilic COPD was not uncommon. In Thai COPD patients, parasitic infestation was not the main reason for high blood eosinophil. Thus, stool parasite exam might be omitted for routine COPD care.

## Supplementary information

Supplementary Tables.
