# Supplementary material for: Eosinophilia and parasitic infestations in patients with chronic obstructive pulmonary disease
Source: Sci Rep. 2020 Jul 27;10:12490. doi: 10.1038/s41598-020-69541-7 (PMC7385115; doi:10.1038/s41598-020-69541-7)
Supplement: Supplementary file 1 — Supplementary Tables. [file 41598_2020_69541_MOESM1_ESM.docx]

**TITLE PAGE**

**Title:** Eosinophilia and Parasitic Infestations in Patients with Chronic Obstructive Pulmonary Disease

**Short running title:** Eosinophilia in COPD

**Authors:** Narongkorn Saiphoklang, M.D.^1,^ * Chanya Chomchoey, M.D.^1^

**Institutional affiliation:** ^1^Division of Pulmonary and Critical Care Medicine, Department of Internal Medicine, Faculty of Medicine, Thammasat University, Thailand

**Corresponding author:** Narongkorn Saiphoklang, M.D.*

Division of Pulmonary and Critical Care Medicine, Department of Internal Medicine, Faculty of Medicine, Thammasat University, 99/209 Paholyotin Road, Klong Luang, Pathum Thani, Thailand 12120, Tel & Fax: +6629269793, e-mail: [m_narongkorn@hotmail.com](mailto:m_narongkorn@hotmail.com)

**Table S1.** Comparison in spirometric data between baseline and 3-month follow-up

| **Variables** | **Baseline** | **3-month follow-up** | **P-value** |
| --- | --- | --- | --- |
| Post-bronchodilator FVC, L | 2.39±0.79 | 2.51±0.84 | 0.040 |
| Post-bronchodilator FVC, % predicted | 80.03±19.48 | 84.56±21.72 | 0.017 |
| FVC change after bronchodilator, % | 9.22±13.44 | 7.40±11.32 | 0.366 |
| Post-bronchodilator FEV_1,_ L | 1.42±0.60 | 1.50±0.62 | 0.025 |
| Post-bronchodilator FEV_1_, % predicted | 66.00±27.42 | 70.94±28.42 | 0.005 |
| FEV_1_ change after bronchodilator, % | 11.51±12.00 | 9.19±10.41 | 0.098 |
| Post-bronchodilator FEV_1_/FVC, % | 57.66±11.68 | 58.47±12.55 | 0.183 |
| Post-bronchodilator FEV_1_/FVC, % predicted | 82.03±17.12 | 83.27±18.32 | 0.312 |
| Post-bronchodilator FEF_25_ -_75,_ L | 0.68±0.40 | 0.78±0.52 | 0.236 |
| Post-bronchodilator FEF_25_-_75,_ % predicted | 31.13±16.58 | 35.33±21.54 | 0.214 |
| FEF_25_-_75_ change after bronchodilator, % | 18.46±29.00 | 17.54±27.16 | 0.844 |

Data shown as mean ± SD, ANOVA was used for comparison of 3 visits.

FVC=forced vital capacity; L=liter; FEV1=forced expiratory volume in 1 second; FEF_25-75_=forced expiration flow rate at 25-75% of forced vital capacity

**Table S2.** Comparison in functional assessment between baseline and 3-month follow-up

| **Variables** | **Baseline** | **3-month follow-up** | **P-value** |
| --- | --- | --- | --- |
| MMRC | 1.58±0.81 | 1.56±0.81 | 0.741 |
| CAT | 9.24±4.93 | 8.52±5.61 | 0.052 |
| 6MWD | 327.62±132.52 | 339.90±138.60 | 0.105 |

Data shown as mean ± SD

6MWD=6-minute walking distance; FEV_1_=forced expiratory volume in 1 second; mMRC=Modified Medical Research Council; CAT=COPD Assessment Test

**Table S3.** Correlation between blood eosinophil count and spirometric data at baseline

| **Parameters** | **Correlation coefficient (r)** | **P-value** |
| --- | --- | --- |
| Post-bronchodilator FVC, L | 0.238 | 0.041 |
| Post-bronchodilator FVC, % predicted | 0.221 | 0.059 |
| FVC change after bronchodilator, % | 0.128 | 0.279 |
| Post-bronchodilator FEV_1,_ L | 0.265 | 0.023 |
| Post-bronchodilator FEV_1,_ % predicted | 0.151 | 0.200 |
| FEV_1_ change after bronchodilator, % | 0.125 | 0.290 |
| Post-bronchodilator FEV_1_/FVC, % | 0.168 | 0.155 |
| Post-bronchodilator FEF_25_-_75,_ L | 0.212 | 0.076 |
| Post-bronchodilator FEF_25_-_75,_ % predicted | 0.138 | 0.250 |
| FEF_25_-_75_ change after bronchodilator, % | 0.167 | 0.165 |

FVC=forced vital capacity; L=liter; FEV_1_=forced expiratory volume in 1 second; FEF_25-75_=forced expiration flow rate at 25-75% of forced vital capacity

**Table S4.** Correlation between blood eosinophil count and functional assessment at baseline

| **Parameters** | **Correlation coefficient (r)** | **P-value** |
| --- | --- | --- |
| mMRC | -0.128 | 0.277 |
| CAT | -0.056 | 0.636 |
| 6MWD | 0.133 | 0.265 |

mMRC=Modified Medical Research Council; CAT=COPD Assessment Test; 6MWD=6-minute walking distance
